# Stressors Facing Home-Based Primary Care Providers

**DOI:** 10.3390/geriatrics4010017

**Published:** 2019-01-28

**Authors:** Katherine O’Brien, Sara Bradley, Vanessa Ramirez-Zohfeld, Lee Lindquist

**Affiliations:** Division of General Internal Medicine and Geriatrics, Department of Medicine, Northwestern University Feinberg School of Medicine, Chicago, IL 60611, USA; sara.bradley@northwestern.edu (S.B.); vanessa-ramirez-0@northwestern.edu (V.R.-Z.); lal425@northwestern.edu (L.L.)

**Keywords:** home-based primary care, home care medicine, homebound

## Abstract

The numbers of homebound patients in the United States are increasing. Home-based primary care (HBPC) is an effective model of interdisciplinary care that has been shown to have high patient satisfaction rates and excellent clinical outcomes. However, there are few clinicians that practice HBPC and clinicians that do face additional stressors. This study sought to better understand the stressors that HBPC providers face in caring for homebound patients. This was a cross-sectional qualitative survey and analysis of HBPC providers. Responses were categorized into four themes: The patient in the home setting, caregiver support, logistics, and administrative concerns. This research is the first to analyze the stressors that providers of HBPC face in serving the needs of complex homebound patients. Awareness and attention to these issues will be important for the future sustainability of home-based primary care.

## 1. Introduction

It is estimated that there are between 3–4 million homebound patients in the United States, and these numbers are expected to increase. Homebound patients are often medically complex, with multiple chronic co-morbidities, frailty, functional impairments, and complex social situations. Approximately 50% of Medicare costs come from roughly 5% of the Medicare population, which encompasses many of these homebound patients [1]. Currently, many of these patients have inconsistent access to traditional office-based care or rely on emergency rooms or even caregivers as their major source of medical care. Home-based primary care (HBPC) has been shown to be an effective way to provide care to complex homebound patients and help them remain in their homes. However, there is currently a major shortage of clinicians providing this type of care. From 2012–2013, there were 1.7 million home visits to Medicare beneficiaries, completed by about 5000 clinicians. Of these, only 475 providers made 1000 or more home visits in 2012 [2]. HBPC is an interdisciplinary model, focusing on comprehensive care in the home for long-term, chronic, complex conditions. The Veterans Administration (VA) has a large HBPC practice. This HBPC program has been shown to have high patient satisfaction rates, as well as decreased hospital bed days of care, nursing home bed days of care, and readmissions [3]. An analysis of another large academic HBPC practice showed similar outcomes, with decreased costs to Medicare, hospitalizations, Emergency Department (ED) visits and Skilled Nursing Facility (SNF) admissions [4]. In the 1980s and 1990s, the number of house calls decreased precipitously, but since that time there has been an increase in the number of house calls performed [5]. Furthermore, HBPC is not only gaining traction in the United States but has also begun to grow internationally as well [6]. Unfortunately, the number of patients that could benefit from HBPC far exceeds the number of clinicians performing house calls [2]. Although the outcomes of HBPC have been shown to be good, and the number of patients who could benefit from the provision of HBPC is increasing, few clinicians are providing HBPC. Additionally, clinicians practicing HBPC face stressors unique to the field. Work focused on home hospice nurses has sought to elucidate stressors facing the nursing staff. Stressors such as collaboration with other practitioners, uncertainty in diagnosis, emotional labor, and workload have all been found to be sources of stress in the hospice setting [7]. To our knowledge, there are no studies focused on stressors in home-based primary care. Therefore, we sought to better understand the stressors that HBPC providers face in caring for homebound patients by conducting a cross-sectional qualitative survey of HBPC providers.

## 2. Methods

A survey was electronically mailed to health care providers who perform home-based primary care and were faculty members at either a Home Centered Care Institute or a Home Care Center of Excellence (Northwestern University, Cleveland Clinic, Mount Sinai (NYC), MedStar Health, University of Pennsylvania, University of Arizona, University of Arkansas, University of California, San Francisco). These centers were chosen as they have been selected as centers with national leaders and programs of excellence in HBPC. All of the centers chosen were affiliated with large academic institutions and provided structured educational courses to others interested in HBPC. Participation in the survey was voluntary. The survey was reviewed by the Northwestern University Institutional Review Board and was considered to be exempt. The survey was sent to participants in the spring of 2017 and consisted of three questions: “In your role as a home-based primary care professional, what are 3–5 top sources of stress you encounter in your day-to-day work—both inside the patient home or from the “back office” perspective?”, “What are 3–5 key attributes/job behaviors that are most important when hiring a HBPC clinical provider/team member?”, and “What are 3–5 key attributes/job behaviors that are most important when hiring a HBPC practice management team manager?” Participants were given space to answer these questions in their own words. Participants had approximately one month to complete and return the survey; all of the surveys received by the deadline were included in the analysis. All of the surveys were anonymous and the coders were blinded. Participants did not receive compensation for their participation. Survey results were then analyzed, using content and constant comparative techniques, through which the coders independently assessed participant responses for focal themes, before convening to compare and compile their findings to create a preliminary list of categories and major themes. The coders then organized the content into an overarching categorical system. From these overarching categories, the coders reached an agreement on themes that were particularly relevant [8,9,10].

## 3. Results

Of the 28 surveys mailed, 21 surveys were returned completed (75% return rate). The responses were provided by physicians (n = 13), nurse practitioners (n = 2), social workers (n = 1), and practice managers (n = 4). There was one provider of unknown discipline. Other demographic data about the respondents was not collected. Responses to the question “What are the top sources of stress in your day-to-day work?” were categorized into four themes by reviewers: The patient in the home setting, caregiver support, logistics, and administrative concerns.

### 3.1. The Patient in the Home Setting 

Homebound patients are more complex and have large numbers of co-morbidities, with constant needs. The providers cited difficulties with managing frequent requirements for urgent needs management and balancing these needs with scheduled visits. There are also issues with uncertainty in management, as providers often do not have easy access to specialists or diagnostic testing to complete a full diagnostic workup. One provider listed “*no specialist backup*”, learning how to “*address urgent needs*”, and “*living with diagnostic uncertainty*” as frequent stressors. In addition, providers often encounter patients with significant mental health issues, including depression and anxiety. Finally, issues personal to the provider were addressed in the responses, such as insect infestations and personal safety, that cause stress.

### 3.2. Caregiver Support

With homebound patients needing assistance in the home, the lack of adequate caregiver support was cited as a stressor to HBPC providers. Even when adequate support is present in the home, primary caregivers are not often easily accessible to providers, making it difficult to provide education, counseling, and planning. One provider shared his concern that “*more caregivers are afraid to take time off to be in the home during the visit, the aide may be the one giving information.*” Conversely, when unable to understand or follow instructions (e.g., due to inadequate health literacy), caregivers can become a stressor to HBPC providers. Additionally, HBPC providers felt stressed when there were family conflicts or interfamilial disagreements.

### 3.3. Logistical Concerns

In performing home visits, HBPC providers cited several different logistical issues causing stressors in their practice, some of which are unique to HBPC. Some of these difficulties were related to how to best perform visits which include planning a schedule, geographic reach, and time management. With a non-traditional model of care, issues regarding patient loads, such as persistently long waiting lists and the increased need to focus on patient recruitment, were cited as stressors. One provider discussed this concern by stating: “*The waitlist*–*we always have one, mostly due to workforce shortage.*” Providers described scheduling difficulties, “*including locational efficiency and acuity*” and “*geographically disbursed clients*”. In addition, there were unique stressors related to team dynamics, including the aligning of staff as a team, the training and education of staff to perform HBPC, and finally, the recruitment and retention of staff. One provider aptly noted that many who work in HBPC do not always have experience in the field, requiring the “*training of staff to understand the unique needs of those with home-limiting illnesses.*”

### 3.4. Administrative Duties 

Finally, there were various administrative stressors noted. Some stressors were similar to those faced by clinicians from all disciplines; these included balancing paperwork with patient care, dealing with electronic health records, audits, and compliance with various mandates and guidelines. One provider described “*ridiculous amounts of bureaucratic hurdles to get things done.*” Monetary concerns regarding reimbursement and funding requirements, as well as developing and maintaining a fiscally responsible business model, represented challenges to HBPC. For example, one provider listed a top stressor as “*inadequate funding for complex work performed.*”

## 4. Discussion

In this qualitative analysis, we identified four themes characterizing the stressors that HBPC clinicians encounter in their day-to-day care of homebound patients, including the patient in the home setting, caregiver support, logistical concerns, and administrative duties.

Ultimately, due to the high number of homebound patients, more providers that are skilled in practicing HBPC are needed [2]. Understanding, and ultimately attempting to mitigate some of the stressors unique to this type of practice, may be a way to help further the workforce in HBPC. Several strategies could be considered moving forward. Providers cited uncertainty in management, with a lack of access to specialty and diagnostic testing to complete a full diagnostic workup, as a stressor. This may begin to improve as technology advances, allowing for increased access in the home (i.e., portable ultrasounds and smartphone-based electrocardiograms) [11,12,13]. In addition, creating partnerships with specialty care could allow HBPC physicians to have at least some indirect assistance when needed. Meanwhile, logistical issues are relatively unique to HBPC. The recruitment of excellent administrative support, attempts at the organization of the visits by geographic area, and attempting to enable providers to have smaller territories within a larger geographic area are some strategies to help offset logistical issues, albeit time-consuming. Making use of newer public transportation resources, such as smartphone-based commuter apps, could decrease the time required to move between visits and allow providers to use the travel time for documentation or phone calls. In recent years, there have been strides forward for the financing of HBPC. For example, Independence at Home is a Medicare demonstration in which practices engaged in shared savings based on lowering the expenses for Medicare. On average, practices saved $3070 per beneficiary in the first year, with overall savings of about $25 million, much of which will be returned to practices [14]. Ongoing collaboration with governmental agencies and other insurers, as well as fundraising and the utilization of grants, will ensure that HBPC can be financially feasible.

While this study sheds light on some of the stressors faced by HBPC providers, there are some limitations to our study. Firstly, the results encompass a small sample size, although this sample size represents a group of clinicians performing large numbers of home visits per year and the overall sample size of clinicians practicing HBPC is small. At the same time, the responses provided were from practices associated with large, primarily urban-based academic medical centers, which may face unique stressors; it would be useful going forward to have an assessment of those practicing in the community or in more rural areas. Finally, as participants were asked to provide 3–5 stressors they face when providing HBPC, it is plausible that there were other more minor stressors that were not captured in this survey.

We identified significant sources of stress unique to HBPC providers in their day-to-day care of homebound patients. While some similarities exist with traditional primary care providers (e.g., balancing documentation with patient care) [15], several unique stressors, including logistics (e.g., driving to multiple locations) and those specific to the home (e.g., insect infestations), were apparent. Caregiver support also featured as an important stressor, which is unsurprising given the frequent dependence of complex homebound patients on others [16]. As financial support/reimbursements for HBPC providers was cited as a major stressor, ongoing future policy reform targeting this aspect could improve health care spending, as HBPC has been shown to be cost-effective in complex patients [1,3,4,5].

## 5. Conclusions

Home-based primary care has been shown to produce cost savings and is associated with high-quality care and patient satisfaction. As the population of complex homebound patients continues to grow, this model of care will become increasingly important. Ultimately, this research is the first to analyze the stressors that providers of HBPC face in serving the needs of complex homebound patients. Awareness and alleviation of these stressors may facilitate more providers entering into and remaining as HBPC providers.

## References

[1] Leff B., Carlson C.M., Saliba D., Ritchie C. (2015). The invisible homebound: Setting quality-of-care standards for home-based primary and palliative care. Health Aff..

[2] Yao N., Ritchie C., Camacho F., Leff B. (2016). Geographic Concentration of Home Based Medical Care Providers. Health Aff..

[3] Beales J.L., Edes T.E. (2009). Veteran’s Affairs home based primary care. Clin. Geriatr. Med..

[4] De Jonge K.E., Jamshed N., Gilden D., Kubisiak J., Bruce S.R., Taler G. (2014). Effects of Home-Based Primary Care on Medicare Costs in High-Risk Elders. J. Am. Geriatr. Soc..

[5] Schuchman M., Fain M., Cornwell T. (2018). The Resurgence of Home-Based Primary Care in the United States. Geriatrics.

[6] McGregor M.J., Cox M.B., Slater J.M., Poss J., McGrail K.M., Ronald L.A., Sloan J., Schulzer M. (2018). A before-after study of hospital use in two frail populations receiving different home-based services at the same time in Vancouver, Canada. BMC Health Serv. Res..

[7] Tunnah K., Jones A., Johnston R. (2012). Stress in hospice at home nurses: A qualitative study of their experiences of their work and wellbeing. Int. J. Pall Nurs..

[8] Charmaz K., Gubrium J.F., Holsteins J.A. (2001). Qualitative interviewing and grounded theory analysis. Handbook of Interview Research: Context and Method.

[9] Glaser B.G., Strauss A.L. (1967). The Discovery of Grounded Theory: Strategies for Qualitative Research.

[10] Strauss A., Corbin J. (1990). Basics of Qualitative Research: Grounded Theory Procedures and Techniques.

[11] Liu L., Stroulia E., Nikolaidis I., Miguel-Cruz A., Rios Rincon A. (2016). Smart homes and home health monitoring technologies for older adults: A systematic review. Int. J. Med. Inform..

[12] Garabelli P., Stavrakis S., Po S. (2017). Smartphone-based arrhythmia monitoring. Curr. Opin. Cardiol..

[13] Mariani P.J., Setla J.A. (2017). Palliative ultrasound for home care hospice patients. Acad. Emerg. Med..

[14] Kinosian B., Taler G., Boling P., Gilden D. (2016). Projected Savings and Workforce Transformation from Converting Independence at Home to a Medicare Benefit. J. Am. Geriatr. Soc..

[15] Riley R., Spiers J., Buszewicz M., Taylor A.K., Thornton G., Chew-Graham C.A. (2018). What are the sources of stress and distress for general practitioners working in England? A qualitative study. BMJ Open.

[16] Stall N., Nowaczynski M., Sinha S.K. (2014). Systematic review of outcomes from home-based primary care programs for homebound older adults. J. Am. Geriatr. Soc..

